# Celastrol ameliorates atherosclerosis by inhibiting TLR4/STAT3/NLRP3-mediated macrophage pyroptosis

**DOI:** 10.1186/s13020-026-01430-z

**Published:** 2026-05-28

**Authors:** Yaning Shi, Ziyi Li, Qizhi Liu, Yun Qiu, Junpeng Long, Qiong Yang, Chanjuan Zhang, Yongzhen Gong, Li Qin

**Affiliations:** 1 Laboratory of Stem Cell Regulation With Chinese Medicine and Its Application, Academy of Chinese Medical Sciences, Hunan University of Chinese Medicine, Changsha, 410208 Hunan China; 2 School of Pharmacy, Hunan University of Chinese Medicine, Changsha, 410208 Hunan China; 3 School of Traditional Chinese Medicine, Hunan University of Chinese Medicine, Changsha, 410208 Hunan China; 4 Institutional Key Laboratory of Vascular Biology and Translational Medicine in Hunan Province, Hunan University of Chinese Medicine, Changsha, 410208 Hunan China

**Keywords:** Celastrol, Atherosclerosis, Macrophage, Pyroptosis, TLR4/STAT3/NLRP3 pathway

## Abstract

**Background:**

Atherosclerosis is a chronic inflammatory disease characterized by plaque formation. Macrophage pyroptosis critically contributes to the formation and expansion of necrotic core as well as the instability of atherosclerotic plaque. Celastrol is a pentacyclic triterpene derived from the root of *Tripterygium wilfordii* Hook F, which has powerful anti-inflammatory properties. This study aimed to explore the effect and mechanism of celastrol in preventing atherosclerosis by regulating macrophage pyroptosis.

**Methods:**

An atherosclerotic mouse model in vivo and a macrophage pyroptosis model induced by ox-LDL in vitro were established successfully. The cell viability was examined by CCK-8. The role of pyroptosis was detected by transmission electron microscopy, LDH assay, and PI staining. HE staining assay was applied to evaluate the morphology of vascular tissues. The degree of lipid accumulation in plaques was measured using Oil Red O staining. The collagen content of plaques was detected using Masson staining. Western blot analysis, RT-qPCR, immunofluorescence staining, and immunohistochemistry were used to determine the levels of key proteins related to pyroptosis in macrophages. The underlying target of celastrol was investigated by CETSA and SPR assays.

**Results:**

Ox-LDL effectively activated macrophage pyroptosis, which was markedly inhibited by celastrol in vitro. Consistently, celastrol suppressed atherosclerotic plaque progression and instability in apoE^−/−^ mice by reducing macrophage pyroptosis in vivo. Mechanistically, celastrol could reduce the phosphorylation and nuclear translocation of STAT3 by targeting and inhibiting TLR4, thereby reducing the activation of NLRP3 inflammasomes and preventing macrophage pyroptosis.

**Conclusion:**

The present study demonstrates for the first time that celastrol prevents macrophage pyroptosis by inhibiting TLR4/STAT3/NLRP3 signaling pathway, ultimately alleviating atherosclerosis. These findings indicate that celastrol can serve as a potential therapeutic drug for atherosclerosis.

## Introduction

Atherosclerosis is a chronic inflammatory disease and a principal contributor to major cardiovascular diseases, including ischemic heart disease, ischemic stroke, peripheral arterial disease and so on [1]. Pathologically, it is characterized by the formation of atherosclerotic plaque in large or medium-sized arteries [2, 3]. In the early stage of atherosclerosis, low-density lipoprotein particles accumulate in the intima, which can undergo oxidative and other modifications that acquire pro-inflammatory and immunogenic properties. Circulating monocytes adhere to endothelial cells and migrate into the subendothelial space under adhesion molecules and chemokines guidance, which can mature into macrophages and subsequently take in lipoprotein particles to transform into foam cells that is a characteristic of atherosclerotic plaques [4, 5]. In the progressing plaque, the death of macrophage-derived foam cells releases lipids, which stores in the center of plaque, forming a necrotic core and exacerbating the instability of the plaque [6]. The unstable plaques of atherosclerosis are prone to rupture, exposing the substances of the interior of the plaque to the blood chamber [2]. The thrombogenic substances in the core of the plaques can trigger thrombosis, blocking the blood vessels [2].

Pyroptosis is a pro-inflammatory form of programmed cell death mediated by inflammasome activation, characterized by the formation of plasma membrane pores mediated by gasdermin [7, 8]. Among various inflammasomes, the nucleotide-binding oligomerization domain-like receptor family pyrin domain containing 3 (NLRP3) inflammasome is the most studied, and its close relationship with atherosclerosis has been widely studied [9]. Upon activation, NLRP3 recruits apoptosis-associated speck-like protein containing a caspase recruitment domain (ASC) and the precursor of caspase-1 (pro-caspase-1), forming NLRP3 inflammasomes, which subsequently induce caspase-1 activation [10, 11]. Activated caspase-1 (representing cleaved-caspase-1) cleaves pro-interleukin-1β (pro-IL-1β) and pro-IL-18 into mature IL-1β and IL-18 [12]. Meanwhile, caspase-1 can also cleave the full-length gasdermin D (GSDMD) into its N-terminal cleavage fragment (N-GSDMD), which forms membrane pores, facilitates release of inflammatory factors, cause cell swelling, and eventually leads to pyroptosis [11]. Macrophage pyroptosis plays a crucial role in the development of atherosclerosis. Within atherosclerotic lesions, macrophage pyroptosis can expand the necrotic lipid core, amplify inflammation, and aggravate plaque instability [11, 13]. During the progression of atherosclerosis, inflammation drives the formation of foam cells, which in turn release pro-inflammatory cytokines, resulting in a vicious cycle [14]. The characteristic of vulnerable plaques is the accumulation of dead cells, of which up to 50% are macrophages [13]. Plaque instability increases the risk of rupture in advanced atherosclerosis, resulting in serious cardiovascular events. Consequently, inhibition of macrophage pyroptosis has emerged as a therapeutic strategy for preventing the progression and instability of atherosclerotic plaques, while its regulatory mechanism is still not entirely explored.

Traditional Chinese medicine and its active ingredients have been proved to have a beneficial effect on the treatment of atherosclerosis [15]. Celastrol (CeT) is a pentacyclic triterpene derived from the root of *Tripterygium wilfordii* Hook F (TwHF, also known as “Thunder God Vine”) [16]. It exhibits a variety of pharmacological effects, including anti-inflammatory, immunosuppressive, antioxidant, vascular protection, as well as antitumor activities [17–19]. There is growing evidence that celastrol attenuates the inflammatory diseases by inhibiting pyroptosis [20, 21]. Given that macrophage pyroptosis is involved in the development of atherosclerosis, we proposed that celastrol may prevent atherosclerosis by inhibiting macrophage pyroptosis.

## Materials and methods

### Materials

Celastrol (C0869), phorbol 12-myristate 13-acetate (PMA, P1585), monophosphoryl lipid A (L6638) and DAPI (D9542) were the products of Sigma-Aldrich (St. Louis, MO, USA). Oxidized low-density lipoprotein (ox-LDL, YB-002) was purchased from Yiyuan Biotechnology (Guangzhou, China). Cell counting kit-8 (CCK-8, K1018) was purchased from APExBIO (Houston, Texas, USA). Lactate dehydrogenase (LDH) assay kit (C0016), BCA assay kit (P0012) and Alexa Fluor 488-labeled Goat Anti-Rabbit IgG (H + L) (A0423) were obtained from Beyotime Institute of Biotechnology (Shanghai, China). PVDF membrane (3010040001) and ECL plus kit (WBKLS0500) were obtained from Millipore Corporation (Billerica, MA, USA). Antibodies against NLRP3 (68102-1-Ig), IL-1β (26048-1-AP), toll-like receptor 4 (TLR4, 19811-1-AP), signal transducer and activator of transcription 3 (STAT3, 10253-2-AP), Lamin B1 (12987-1-AP), GAPDH (10494-1-AP), α-Tubulin (66031-1-Ig), the peroxidase‐conjugated anti‐rabbit (SA00001-2), and anti-mouse (SA00001-1) secondary antibodies were obtained from Proteintech (Chicago, Illinois, USA). Antibody against N-GSDMD (AB215203) was the product of Abcam (Cambridge, MA, USA). Antibodies against cleaved caspase-1 (cle-caspase-1, AF4005) and p-STAT3 (AF3293) were purchased from Affinity (Melbourne, Australian). TLR4 protein (10146-H08B) was purchased from Sino Biological (Beijing, China). Hoechst 33342/propidium iodide (PI) (CA1120) double staining kit and Oil Red O (G1262) were obtained from Solarbio (Beijing, China). Trizol reagent (R1030) was produced by Applygen Technologies Inc. (Beijing, China). cDNA synthesis kit (6111A) and qPCR kit (RR047A) were purchased from Takara (Kyoto, Japan). Nigericin (HY-127019) and colivelin TFA (HY-P1061A) were obtained from MedChemExpress (New Jersey, USA). Atorvastatin calcium (ATV, 100,590) was purchased from National Institutes for Food and Drug Control (Beijing, China). HRP-conjugated secondary antibody and DAB peroxidase substrate solution (PV-8000) were purchased from ZSGB-BIO (Beijing, China).

### Cell culture

Human acute monocytic leukemia cell line THP-1 was obtained from Cell Bank of the Chinese Academy of Sciences (Shanghai, China). THP-1 cells were cultured in RPMI-1640 medium supplemented with 10% fetal bovine serum, 100 IU/mL penicillin and 100 μg/mL streptomycin streptomycin at 37 ℃ in 5% CO_2_ humidified atmosphere. For differentiation into macrophages, THP-1 cells were treated with 100 nM PMA for 24 h.

### Cell viability assay

Cell viability was determined using CCK-8 assay kit. THP-1 cells were seeded in 96-well plate at a density of 2 × 10^4^ cells/well and differentiated into macrophages by using PMA. After different treatments, 10 μL CCK-8 reagent was added into each well and incubated at 37 °C for 30 min. The optical density (OD) value at 450 nm was detected by using a microplate reader (EL × 800, BioTek, Winooski, VT, USA).

### LDH assay

Briefly, THP-1 cells were cultured into 96-well plates for 24 h. After different treatments, the 120 μl of culture supernatant was transferred to a new 96-well plate. Each well was added with 60 μl working solution, and incubated for 25 min at room temperature in the dark. The absorbance of each group was determined at 490 nm.

### Western blotting

Total proteins were extracted from THP-1 macrophages or aortic tissues by using RIPA lysis buffer containing protease and phosphatase inhibitor. The lysate was centrifuged at 12,000 rpm for 20 min at 4 ℃, and then the supernatant was collected. Protein concentration was quantified using a BCA assay kit. Aliquots of the samples were separated by SDS-PAGE and then transferred to PVDF membranes. After blocking with 5% non-fat milk in TBST for 2 h, the membranes were incubated with primary antibodies overnight at 4 °C. After washing with TBST, the membranes were incubated with secondary antibodies for 2 h at room temperature. The antibody binding content was detected by visualizer (Tanon-5200, Shanghai, China). Protein levels were quantified by Image J.

### Hoechst 33,342/PI staining

Hoechst 33342/PI staining was performed according to the manufacturer’s instructions. After different treatments, THP-1 macrophages were carefully washed twice by cold PBS, then stained with PI (2 μg/mL) and Hoechst33342 (5 μg/mL) at 4 ℃ for 30 min. Then, cells were washed once by cold PBS and observed by using an inverted fluorescence microscope (Olympus, Tokyo, Japan).

### Real-time reverse transcription quantitative polymerase chain reaction (RT-qPCR)

Total RNA was isolated from treated THP-1 macrophages using trizol reagent. cDNA was synthesized using the cDNA synthesis kit, and qPCR was performed using a qPCR kit on a real‐time PCR detection system (CFX96, Bio-Rad). The mRNA expression of NLRP3 was quantified. Data were normalized to the mRNA expression level of GAPDH.

### Immunofluorescent staining

Treated THP-1 macrophages on glass slides were washed thrice by PBS and then fixed with 4% paraformaldehyde for 30 min. The 4% paraformaldehyde was removed and the cells were permeabilized with 0.5% Triton X-100 for 20 min, then washed three times with PBS. Next, the cells were blocked with 3% bovine serum albumin for 2 h at room temperature. Afterwards, the cells were incubated with primary antibody for 1 h at room temperature, followed by incubating with Alexa Fluor 488-conjugated secondary antibody for 1.5 h at room temperature. Ultimately, the cells were incubated with DAPI for 5 min and observed by an inverted fluorescence microscope.

### Cellular thermal shift assay (CETSA)

Briefly, treated THP-1 macrophages were suspended in 1 mL of PBS and divided into six aliquots, followed by heating for 3 min at the indicated temperatures (40℃-65℃). After heating, cells were lysed by three freeze–thaw cycles and lysates were centrifuged at 12,000 rpm for 20 min at 4 ℃. Supernatants were subject to western blotting to detect TLR4 protein levels, with thermal stability reflecting drug-target interaction.

### Surface plasmon resonance (SPR)

SPR assays were performed with a Biacore T200 (Cytiva Sweden AB). The CM5 sensor chip was employed for the immobilization of recombinant human TLR4 protein. The ligand (celastrol) was dissolved for preparation. TLR4 protein was diluted to 50 μg/mL, with an optimal pH of 4.0 determined by pre-coupling. First, carry out chip activation for a duration of 420 s. Then, sodium acetate solution with pH 4.0 was used to flow through the first channel of CM5 chip as a reference, and TLR4 protein diluted with sodium acetate solution with pH 4.0 was used to flow through different channels, coupled to 600 s. After immobilization, residual active sites on the chip surface were blocked, and the resulting coupling signal intensity was recorded. Celastrol was dissolved with PBS with 5% DMSO for a series of concentrations. The test samples were injected at a flow rate of 30 μL/min for 300 s, and different concentrations of celastrol were compared with TLR4 immobilized on the chip. PBS containing 5% DMSO was injected into the channel of CM5 chip at a flow rate of 30 μL/min for 300 s, and the compound were dissociated from the chip, and the binding and dissociation curves of compound and protein were obtained. The SPR detection curves were solvent‑corrected, and reference and buffer subtraction was then performed using Biacore T200 evaluation software to determine the equilibrium dissociation constant (Kd) values for the binding of the test compound to the protein.

### Animals

6–8 weeks male apolipoprotein E deficient (ApoE^−/−^) mice with a C57BL/6 background (Beijing HFK Bioscience Co., Ltd, China) and C57BL/6 mice (Hunan SJA Laboratory Animal Co., Ltd, China) were used in this study. Mice were maintained under a 12-h dark–light cycle with free access to food and water.

### Animal experiments

Mice were randomly divided into seven groups and subjected to different diets for 12 weeks. C57BL/6 mice were fed a normal chow diet served as the control group. ApoE^−/−^ mice in the model group were fed a high-fat diet (HFD, 76.55% ordinary feed, 12% lard, 10% egg yolk powder, 0.2% sodium cholate, 1.25% cholesterol). ApoE^−/−^ mice in the low-, middle-, and high- concentration celastrol groups were fed a HFD and intraperitoneally injected with 0.25, 0.5, 1 mg/kg/2 d celastrol (containing 1% DMSO), respectively. ApoE^−/−^ mice in the vehicle group were fed a HFD and intraperitoneally injected with vehicle. ApoE^−/−^ mice in the ATV group were fed a HFD and received oral medication of 10 mg/kg/2 d ATV. After the experiment, mice were euthanized, and plasma, aorta and heart samples were collected for further analyses.

### Atherosclerosis analysis

After the mice were euthanized, the heart and entire aorta from the proximal ascending aorta to the bifurcation of the iliac artery were immediately dissected and then fixed in 4% paraformaldehyde. Subsequently, the adventitia of aorta was removed carefully. The aorta was unfolded along the longitudinal axis, stained with Oil Red O, and photographed with a digital camera. After dehydration, the heart of mice with upper aortic root was embedded in optimal cutting temperature compound and serial sectioned into 8-μm-thick frozen sections throughout the three aortic valves. Whereafter, the sections were stained with hematoxylin and eosin (HE), Oil Red O and Masson. The lesion areas were detected by Oil Red O staining, the necrotic core contents were detected by HE staining, and the collagen contents were detected by Masson staining. All analyses were quantified using Image-Pro Plus 6.0.

### Immunohistochemical staining

Briefly, the sections of paraffin-embedded tissue underwent dewaxing using xylene, rehydration in a stepwise manner using ethyl alcohol, and antigen retrieval. After a 30 min incubation at room temperature with blocking buffer, the sections were incubated with primary antibodies at room temperature for 2 h. Then, the sections were incubated with HRP-conjugated secondary antibodies at 37 °C for 30 min. Subsequently, the sections were incubated in DAB peroxidase substrate solution for 1 min and then washed with water. Finally, the sections were counterstained with hematoxylin for 2 min and differentiated with a hydrochloric acid/alcohol mixture. After dehydration and mounting, the stained tissue was observed and analyzed under an upright microscope (DM2000, Leica), and quantitative analysis was performed using Image-Pro Plus 6.0 software.

### Statistical analysis

All data are expressed as the mean ± standard deviation (SD) from at least three independent experiments (biological replicates). All statistical analyses among groups were performed by one-way analysis of variance (ANOVA) test with Dunnett’s method. GraphPad Prism 10 was used for statistical analyses. A value of P < 0.05 was considered statistical significance.

## Results

### Celastrol alleviates atherosclerotic plaque formation and enhances plaque stability in ApoE^−/−^ mice

**Fig. 1:**
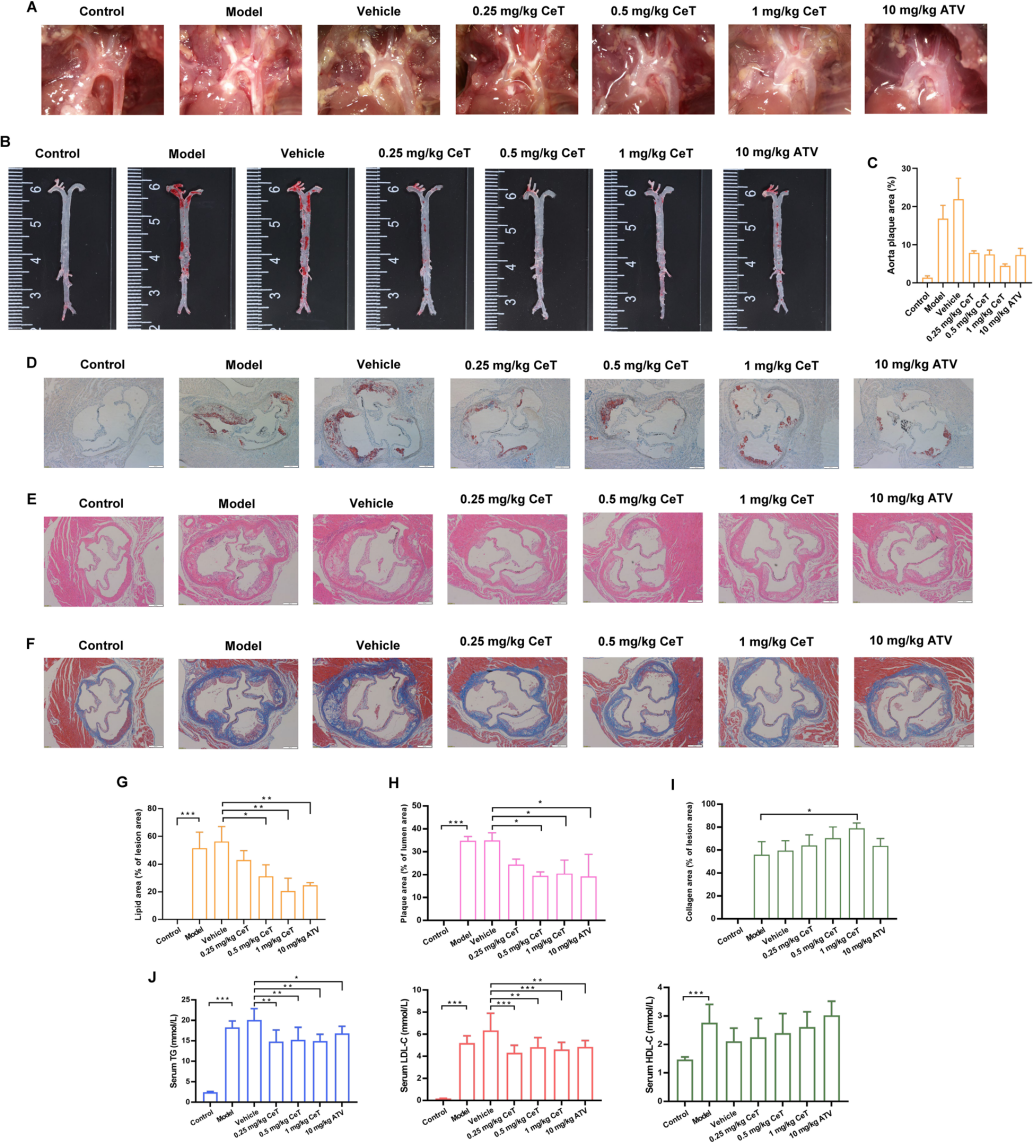
Celastrol alleviates the progression of atherosclerosis in ApoE^−/−^ mice. Animals were divided into the following groups: control group (C57BL/6 mice + normal diet), model group (ApoE^−/−^ mice + HFD), low-, medium-, high-dose celastrol groups (ApoE^−/−^ mice + HFD + 0.25, 0.5, 1 mg/kg celastrol, respectively), vehicle group (ApoE^−/−^ mice + HFD + vehicle), and ATV group (ApoE^−/−^ mice + HFD + ATV). After the experiment, the tissues were harvested. **A** Representative image of white plaques in the aortic arch region of ApoE^−/−^ mice. **B** Representative photos of aortic en face stained with Oil Red O. **C** Quantification of plaque burdens in the entire aorta. **D** Lipid accumulation was indicated by Oil Red O staining in sections of the aortic root (Scale: 200 μm). **E** Atherosclerotic lesions were visible in HE-stained sections of the aortic root (Scale: 200 μm). **F** The content of collagen fibers in the aortic root was determined using Masson staining (Scale: 200 μm, blue indicating collagen fibers). **G** Quantification of Oil Red O staining for the aortic root. **H** Quantification of HE staining for the aortic root. **I** Quantification of Masson staining for the aortic root. **J** The level of TG, LDL-C and HDL-C in serum. Data were expressed as mean ± SD (n = 3). ^*^P < 0.05, ^**^P < 0.01, and ^***^P < 0.001

To evaluate whether celastrol protects against atherosclerosis in vivo, ApoE^−/−^ mice were fed a HFD for 12 weeks and simultaneously received intraperitoneal injection of celastrol or oral gavage of ATV. Herein, ATV was used as a positive control. These results indicated that both celastrol and ATV significantly reduced atherosclerotic plaque area and lipid deposition in the aortic arch regions (Fig. 1A–E, G, H). Given that collagen content is a key factor of plaque stability, we performed Masson staining assay, which revealed that celastrol treatment dramatically increased the content of collagen (Fig. 1F, I). Furthermore, celastrol significantly reduced triglyceride (TG) and low-density lipoprotein cholesterol (LDL-C) levels in serum, while there was no significant difference in high-density lipoprotein cholesterol (HDL-C) level (Fig. 1J). Taken together, the data demonstrate that celastrol attenuates atherosclerotic plaque progression and increases plaque stability in vivo.

### Celastrol inhibits THP-1 macrophage pyroptosis induced by ox-LDL

**Fig. 2:**
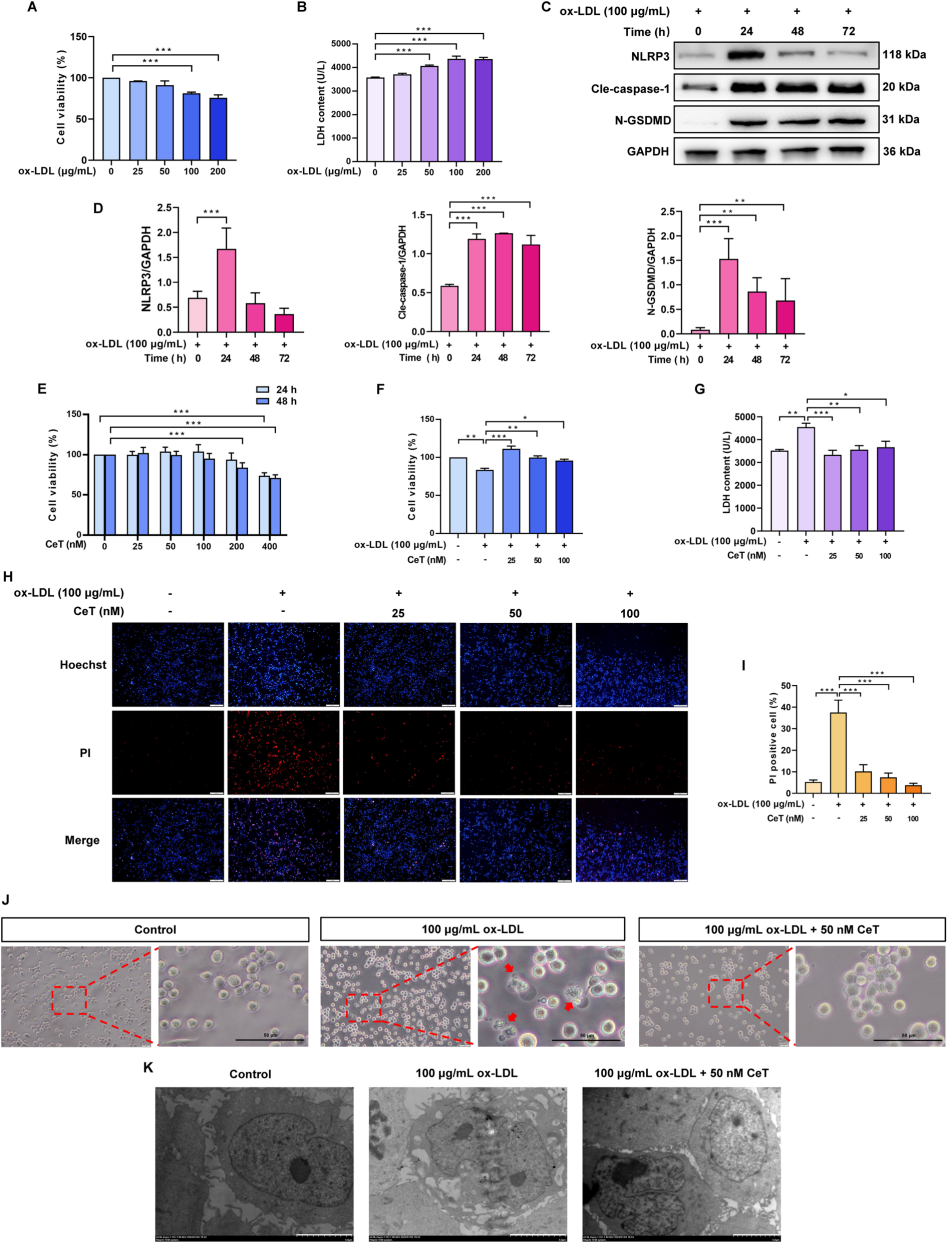
Celastrol attenuates ox-LDL-induced THP-1 macrophage pyroptosis. **A** After differentiation with 100 nM PMA for 24 h, THP‑1‑derived macrophages were treated with 25–200 µg/mL ox‑LDL for 24 h. The cell viability was evaluated by CCK‑8 assay. **B** LDH level was detected. **C**, **D** THP-1 macrophages were treated with 100 µg/mL ox-LDL for 24–72 h. The protein expression of NLRP3, cle-caspase-1, and N-GSDMD was analyzed by western blotting. **E** THP-1 macrophages were treated with 50–200 nM celastrol for 24 h or 48 h and cell viability was detected by CCK-8 assay. **F** THP-1 macrophages were pre-exposed to 25–100 nM celastrol for 1 h, and then supplemented with 100 µg/mL ox-LDL for 24 h co-treatment. Cell viability was assessed using CCK-8 assay. **G** LDH release was measured. **H**, **I** Representative photos and quantification of Hoechst/PI staining (Scale: 200 μm). **J** Representative images of microscope (Scale: 50 μm) showing morphological characteristic of pyroptosis, including cell swelling and membrane blebbing. **K** Representative photos of transmission electron microscope (Scale: 5 μm) showing ultrastructural features of pyroptosis, including membrane pore formation and rupture. Results were represented as mean ± SD (n = 3). ^*^P < 0.05, ^**^P < 0.01, and ^***^P < 0.001

During atherosclerotic plaques progression, ox-LDL can induce macrophage pyroptosis by activating NLRP3 inflammasome, which significantly contributes to plaque destabilization [22]. Firstly, we assessed the effects of ox-LDL on macrophage pyroptosis in vitro. THP-1 monocytes were differentiated into macrophages using 100 nM PMA for 24 h, followed by exposure to 25–200 µg/mL ox-LDL for 24 h or 100 µg/mL ox-LDL for 24–72 h. The data showed that ox-LDL reduced cell viability at concentrations of 100 and 200 µg/mL and increased LDH release at 50–200 µg/mL for 24 h (Fig. 2A, B). Western blot analysis further revealed that treatment with 100 µg/mL ox-LDL for 24 h significantly elevated levels of pyroptosis-related protein, including NLRP3, cle-caspase-1 and N-GSDMD (Fig. 2C, D). Next, the effect of celastrol on the viability of THP-1 macrophages was examined by CCK-8 assay. No obvious toxicity was detected in THP-1 macrophages treated with 50–200 nM celastrol for 24 h and 50–100 nM celastrol for 48 h (Fig. 2E). In order to explore the inhibitory effect of celastrol on macrophage pyroptosis in vitro, THP-1 macrophages were treated with 100 µg/mL ox-LDL for 24 h in the absence or presence of 25–100 nM celastrol. The results revealed that celastrol effectively reversed ox-LDL-induced the reduction of cell viability and the increase of LDH release and PI-positive cells (Fig. 2F–I). Moreover, the typical feature of pyroptosis induced by ox-LDL, such as cell swelling and membrane rupture, were notably ameliorated by celastrol treatment (Fig. 2J, K). These findings suggest that celastrol suppresses THP-1 macrophage pyroptosis induced by ox-LDL.

### Celastrol reduces macrophage pyroptosis by impeding NLRP3 inflammasome activation

**Fig. 3:**
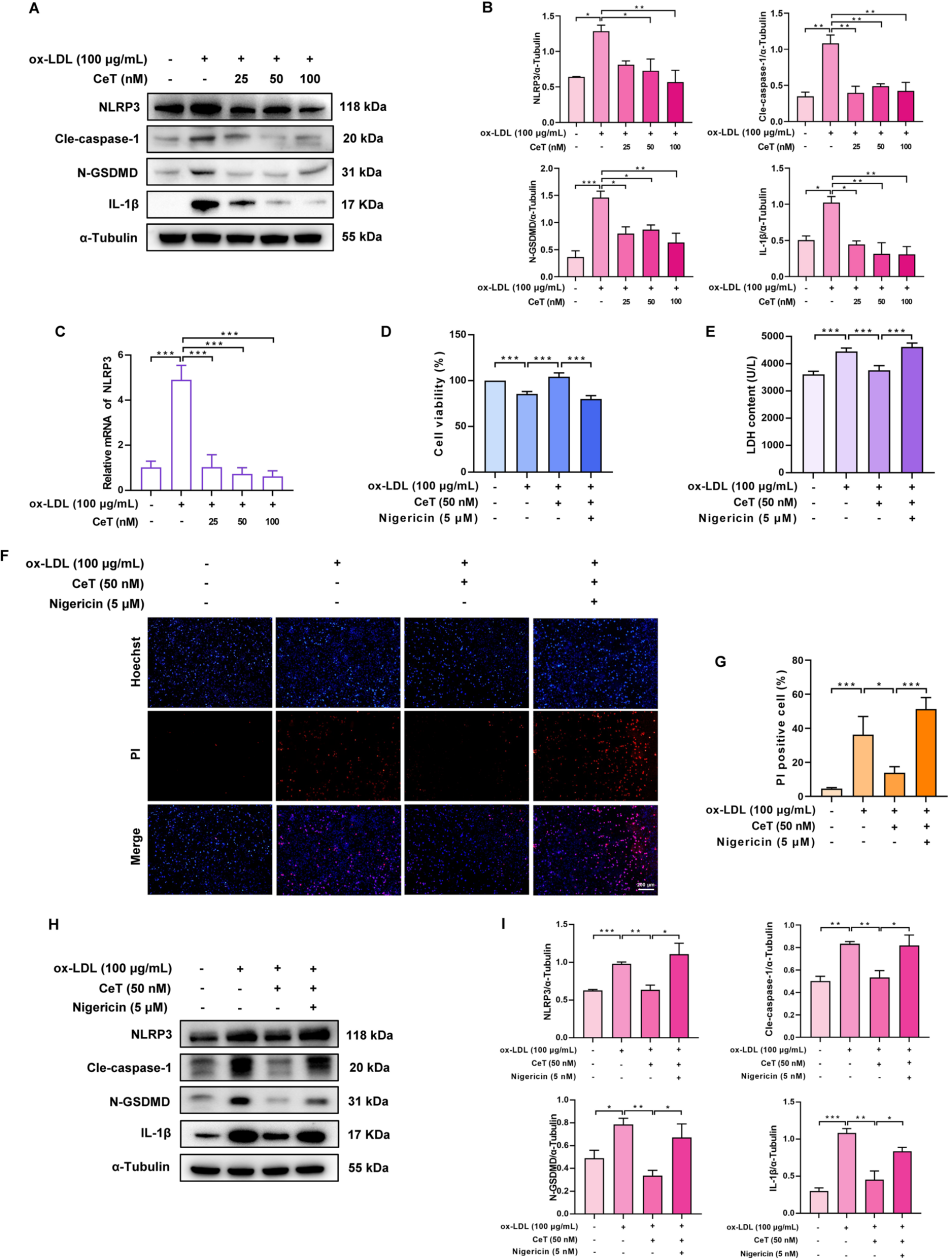
Celastrol decreases THP-1 macrophage pyroptosis by preventing the activation of NLRP3 inflammasome. **A**, **B** The protein level of NLRP3, cle-caspase-1, N-GSDMD, and IL-1β. **C** The mRNA level of NLRP3. **D** THP-1 macrophages were treated with 100 µg/mL ox-LDL in the presence or absence of 50 nM celastrol and 5 μM nigericin for 24 h. CCK-8 assay was employed to evaluate cell viability. **E** LDH level was measured. **F**, **G** Representative photos and quantification of Hoechst/PI staining (Scale: 200 μm). **H**, **I** The expression of NLRP3, cle-caspase-1, N-GSDMD, and IL-1β proteins. Data were shown as mean ± SD (n = 3). ^*^P < 0.05, ^**^P < 0.01, and ^***^P < 0.001

Given that NLRP3 inflammasome activation drives macrophage pyroptosis and IL-1β release [23], we examined the effect of celastrol on NLRP3 inflammasomes. As shown in Fig. 3A–C, ox-LDL treatment upregulated the protein levels of NLRP3, cle-caspase-1, N-GSDMD and IL-1β, while these effects were reversed by celastrol. Next, the NLRP3 activator nigericin was used to explore the role of NLRP3 inflammasome in the celastrol-mediated inhibition of macrophage pyroptosis. It was observed that nigericin could abolish the celastrol-mediated enhancement of cell viability and decrease of LDH release and PI-positive cells (Fig. 3D–G). Additionally, the inhibitory effects of celastrol on the protein levels of NLRP3, cle-caspase-1, N-GSDMD and IL-1β could be reversed by nigericin (Fig. 3H, I). These data indicate that celastrol inhibits macrophage pyroptosis by preventing NLRP3 inflammasome activation.

### Celastrol abrogates macrophages pyroptosis by regulating TLR4 and STAT3

**Fig. 4:**
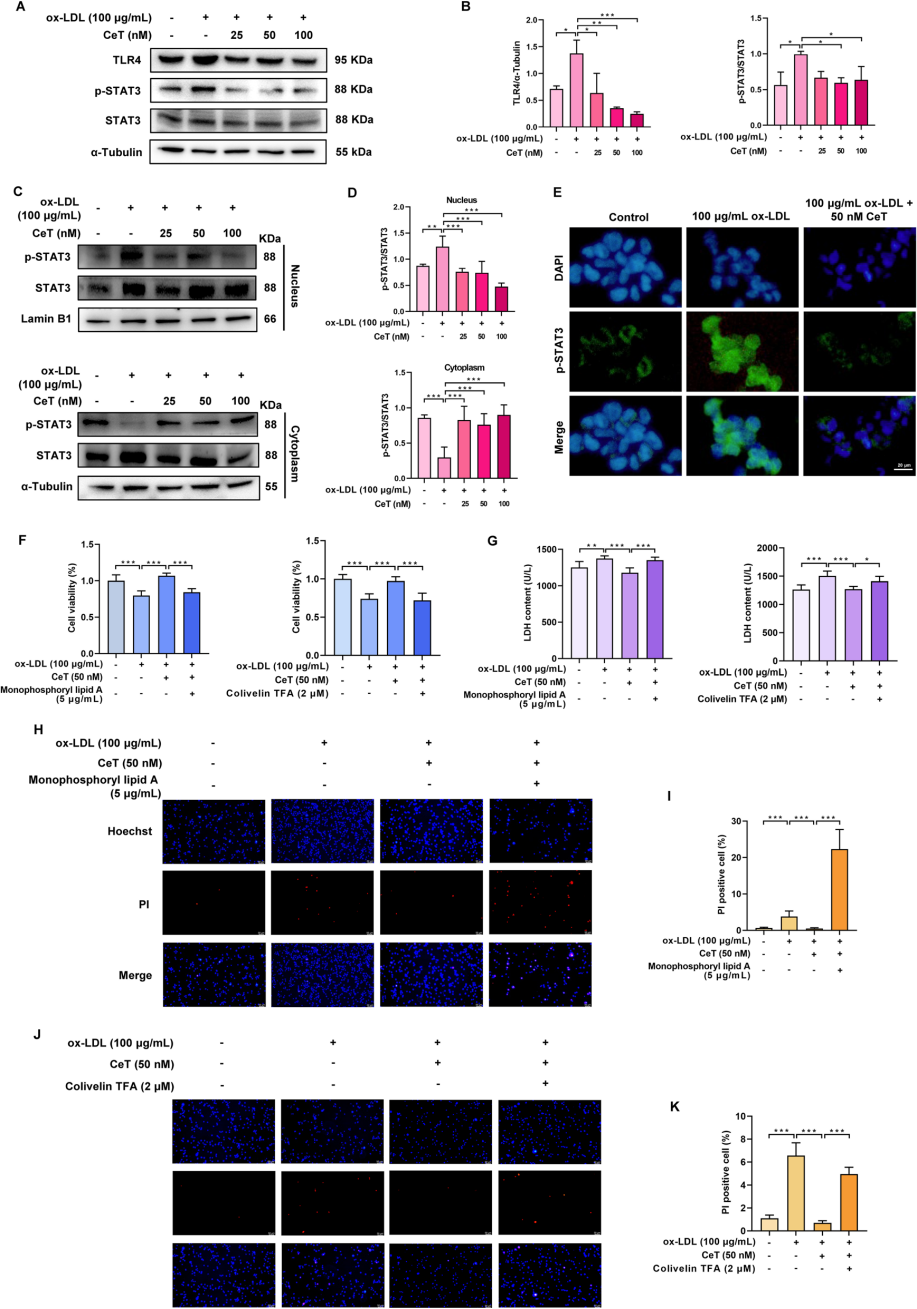
Celastrol suppresses THP-1 macrophage pyroptosis through regulation of TLR4 and STAT3. **A**, **B** The protein level of TLR4, p-STAT3, and STAT3 was evaluated by western blot analysis. **C**, **D** Western blot analysis of the expression levels of p-STAT3 and STAT3 in nucleus and cytoplasm. **E** Immunofluorescence was used to observe the translocation of p-STAT3 (Scale: 20 μm). **F** THP-1 macrophages were treated with 100 µg/mL ox-LDL in the presence or absence of 50 nM celastrol, 5 µg/mL monophosphoryl lipid A and 2 µM colivelin TFA for 24 h. The cell viability was measured by CCK-8 assay.** G** LDH release was measured. **H–K** Representative images and quantification of Hoechst/PI staining (Scale: 50 μm). Data were presented as mean ± SD (n = 3). ^*^P < 0.05, ^**^P < 0.01, and ^***^P < 0.001

It is known that TLR4 and STAT3 are closely related to macrophage pyroptosis [24, 25]. We found that celastrol suppressed ox-LDL-induced upregulation of TLR4 expression and the p-STAT3/STAT3 ratio during THP-1macrophage pyroptosis (Fig. 4A, B). Additionally, celastrol inhibited ox-LDL-induced nuclear translocation of phosphorylated STAT3 (Fig. 4C–E). Using the TLR4 activator monophosphoryl lipid A and STAT3 activator colivelin TFA, we found that activation of TLR4 and STAT3 reversed the suppressive effect of celastrol on macrophage pyroptosis, as evidenced by the reversal of celastrol‑induced improvements in cell viability, reductions in LDH release, and decreases in PI‑positive cell numbers (Fig. 4F–K). Taken together, these data suggest that the inhibition of macrophage pyroptosis by celastrol may be associated with the regulation of TLR4 and STAT3.

### TLR4/STAT3/NLRP3 signaling pathway is involved in the suppressive effect of celastrol on macrophages pyroptosis

**Fig. 5:**
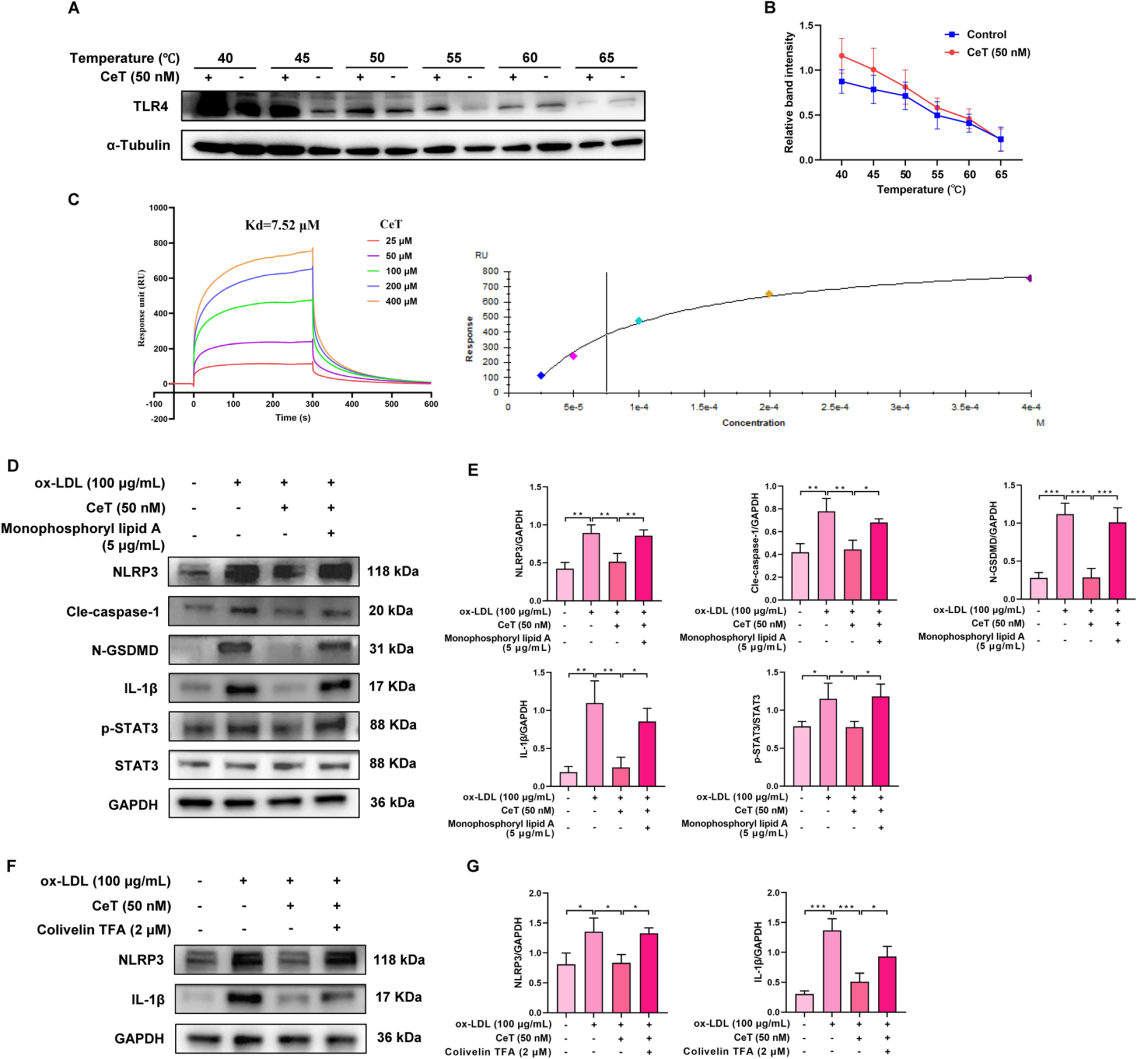
Celastrol reduces THP-1 macrophages pyroptosis by inhibiting TLR4/STAT3/NLRP3 signaling pathway. **A**, **B** The interaction between celastrol and TLR4 was identified by CETSA analysis. **C** The binding affinity between celastrol and TLR4 was evaluated by SPR assay. **D**, **E** The expression of NLRP3, cle-caspase-1, N-GSDMD, IL-1β, p-STAT3, and STAT3 proteins. **F**, **G** The level of NLRP3 and IL-1β proteins. Results were shown as mean ± SD (n = 3). ^*^P < 0.05, ^**^P < 0.01, and ^***^P < 0.001

To explore whether TLR4 is a direct target for celastrol, CETSA and SPR assays were performed. As shown in Fig. 5A, B, celastrol enhanced the thermal stability of TLR4 compared to control group. The Kd value between celastrol and TLR4 was 7.52 μM (Fig. 5C), confirming that celastrol directly binds to TLR4. Notably, the TLR4 activator monophosphoryl lipid A and the STAT3 activator colivelin TFA reversed the inhibitory effect of celastrol on NLRP3 inflammasome activation in THP-1 macrophages, respectively (Fig. 5D–G). Furthermore, monophosphoryl lipid A abrogated the suppressive effects of celastrol on STAT3 phosphorylation (Fig. 5D, E). These findings demonstrate that celastrol inhibits macrophage pyroptosis by blocking the TLR4/STAT3/NLRP3 signaling pathway.

### Celastrol attenuates atherosclerosis by inhibiting the TLR4/STAT3/NLRP3 signaling pathway in vivo

**Fig. 6:**
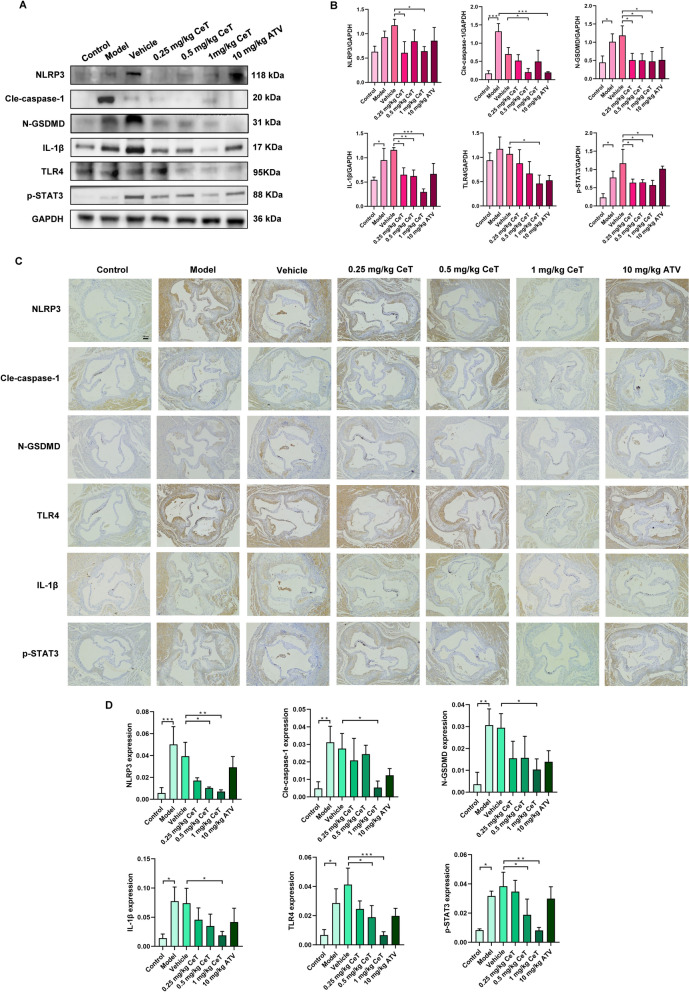
TLR4/STAT3/NLRP3 signaling pathway participates in the suppressive effect of celastrol on atherosclerosis. **A**, **B** The protein expression of NLRP3, cle-caspase-1, N-GSDMD, IL-1β, TLR4, p-STAT3, and STAT3 was assessed by western blot analysis. **C**, **D** Representative images and quantification of immunohistochemistry staining for NLRP3, cle-caspase-1, N-GSDMD, IL-1β, TLR4, and p-STAT3 (Scale: 100 μm). Results were expressed as mean ± SD (n = 3). ^*^P < 0.05, ^**^P < 0.01, and ^***^P < 0.001

To evaluate whether celastrol impedes atherosclerosis in vivo by suppressing pyroptosis via inhibiting TLR4/STAT3/NLRP3 pathway, we conducted experiments in ApoE^−/−^ mice. Key proteins associated with pyroptosis and this signaling pathway were analyzed by western blotting and immunohistochemistry. The results revealed that HFD significantly increased the expressions of TLR4, p-STAT3, NLRP3, cle-caspase-1, N-GSDMD and IL-1β, while these effects were reversed by celastrol treatment in the aortic tissue of ApoE^−/−^ mice (Fig. 6A–D). This finding suggests that celastrol alleviates atherosclerosis by suppressing TLR4/STAT3/NLRP3 signaling pathway.

## Discussion

Atherosclerosis is a chronic inflammatory disease of blood vessels characterized by the narrowing and vulnerability of plaque [26]. Despite the widespread use of statins against atherosclerosis in clinical practice, their long-term toxicity, such as hemorrhagic stroke, new-onset diabetes mellitus, and statin-associated muscle symptoms, highlights the need for other therapeutic strategies [27]. Clinical studies have revealed that residual inflammatory risk was a stronger predictor of future cardiovascular events and death than residual cholesterol risk [28]. Celastrol, as an active ingredient of TwHF, has excellent anti-inflammatory activity that can alleviate various inflammatory diseases, including rheumatoid arthritis, osteoarthritis, gouty arthritis, and inflammatory bowel disease [19]. Our previous studies have shown that celastrol exerts anti-atherosclerotic effects by reducing foam cell formation [17]. It has been reported that pyroptosis is a form of programmed cell death characterized by the release of pro-inflammatory cytokines, which widely occurs in the initiation and development of atherosclerosis [7, 11, 29]. Herein, we originally propose the role and mechanism of celastrol in preventing atherosclerosis by inhibiting macrophages pyroptosis.

The expansion of necrotic core during atherosclerotic progression is a key determinant of plaque fragility and rupture, contributing significantly to acute cardiovascular events [4]. Accumulating studies have shown that macrophage pyroptosis promotes the release of pro-inflammatory cytokines, induces the formation of foam cells, expands plaque necrotic core, as well as accelerates plaque instability in atherosclerotic lesions [7, 29, 30]. Our study demonstrates that celastrol effectively suppresses the formation and progression of plaques, promotes plaque stability, and reduces inflammation by blocking NLRP3 inflammasome activation and macrophage pyroptosis in ApoE^−/−^ mice. Similarly, we in vitro found that celastrol blocks the activation of NLRP3 inflammasome and the release of inflammatory cytokine induced by ox-LDL in macrophages. These data indicated that celastrol delay atherosclerotic progression, which is closely related to the inhibition of macrophage pyroptosis and the reduction of pro-inflammatory cytokine release.

Studies have confirmed that celastrol inhibits inflammatory responses through multiple pathways, including the TLR4, NF-κB, MAPK, and PI3K/Akt signaling pathways [31]. TLR4, a key pattern recognition receptor on the cell surface, plays a pivotal role in pyroptosis. Downregulation of TLR4 signaling attenuates pyroptosis of vascular smooth muscle cell-derived foam cells induced by ox‑LDL, thereby ameliorating atherosclerotic lesions [32]. Moreover, the TLR4 signaling pathway is also involved in exacerbating various diseases, such as spinal cord injury, rheumatoid arthritis, and hepatic ischemia–reperfusion injury, by mediating NLRP3 inflammasome activation and inducing pyroptosis [33–35]. Importantly, our results showed that celastrol directly binds to TLR4 and inhibits the activation of NLRP3 inflammasome by down-regulating TLR4 expression in atherosclerotic plaque in vivo and in ox-LDL-induced THP-1 macrophages in vitro, respectively. However, the underlying mechanism by which TLR4 mediates NLRP3 inflammasome activation requires further exploration. STAT3, as a cytokine-responsive transcription factor, can promote NLRP3 inflammasome activation [36, 37]. Conversely, inactivation of STAT3 can hinder caspase-1 activation and IL-1β secretion induced by NLRP3 inflammasome in primary macrophages [36]. There is evidence that inhibition of the TLR4/STAT3 pathway in macrophages reduces inflammatory responses [38]. In this study, celastrol prevents STAT3 phosphorylation and nuclear translocation both in plaque and in ox-LDL-induced THP-1 macrophages. Furthermore, the STAT3 activator colivelin TFA reverses the inhibitory effect of celastrol on macrophage pyroptosis, while the TLR4 agonist monophosphoryl lipid A reverses celastrol-mediated suppression of macrophage pyroptosis and STAT3 phosphorylation in vitro. Collectively, our data clearly show that celastrol downregulates TLR4 to inhibit STAT3 activation and nuclear translocation, thereby blocking NLRP3 inflammasome activation in macrophages. Nevertheless, several limitations should be mentioned. First, the in vitro experiments used the THP-1 cell line rather than primary macrophages. Second, it remains unclear whether other signaling pathways are involved in celastrol-mediated inhibition of macrophage pyroptosis and whether there are crosstalk or synergistic effects among these pathways. Third, although CETSA and SPR confirmed direct binding between celastrol and TLR4, the binding site or structural basis of this interaction have not been resolved. Additionally, while celastrol holds promise as a therapeutic agent, its known toxicities [39] necessitate further optimization of dosing regimens and drug delivery systems (e.g., nanoformulation) to improve its safety profile for clinical translation.

## Conclusion

**Fig. 7:**
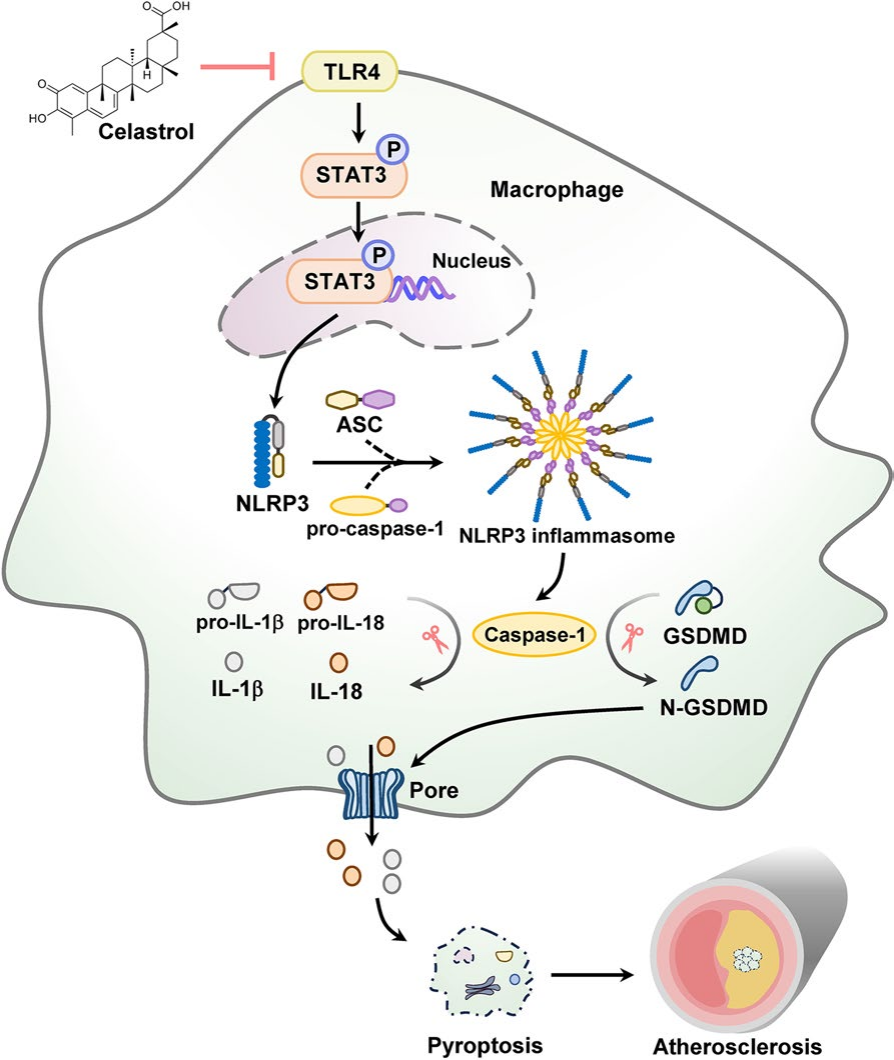
Schematic illustration of the mechanism by which celastrol inhibits atherosclerosis. Celastrol reduces macrophage pyroptosis by suppressing the TLR4/STAT3/NLRP3 pathway, and ultimately preventing atherosclerosis

In conclusion, this study provides solid evidence that celastrol attenuates atherosclerosis and elucidates its underlying mechanism. Our results demonstrate that celastrol impedes macrophage pyroptosis through suppressing TLR4/STAT3/NLRP3 signaling pathway, thereby alleviating atherosclerosis (Fig. 7). Collectively, this work highlights celastrol as a promising therapeutic candidate for the treatment of atherosclerosis.

## Data Availability

The data produced from this study are available from the corresponding author on reasonable request.

## Abbreviations

ApoE^−^^/^^−^: Apolipoprotein E deficient
ASC: Apoptosis-associated speck-like protein containing a caspase recruitment domain
ATV: Atorvastatin calcium
CeT: Celastrol
CCK-8: Cell counting kit-8
CETSA: Cellular thermal shift assay
Kd: Equilibrium dissociation constant
GSDMD: Gasdermin D
HE: Hematoxylin and eosin
HDL-C: High-density lipoprotein cholesterol
HFD: High-fat diet
LDH: Lactate dehydrogenase
LDL-C: Low-density lipoprotein cholesterol
NLRP3: Nucleotide-binding oligomerization domain-like receptor family pyrin domain containing 3
ANOVA: One-way analysis of variance
OD: Optical density
ox-LDL: Oxidized low-density lipoprotein
PMA: Phorbol ester
RT-qPCR: Real-time reverse transcription quantitative polymerase chain reaction
STAT3: Signal transducer and activator of transcription 3
SD: Standard deviation
SPR: Surface plasmon resonance
TLR4: Toll-like receptor 4
TG: Triglyceride
TwHF: *Tripterygium wilfordii* Hook F
